# *Plasmodium* schizogony, a chronology of the parasite’s cell cycle in the blood stage

**DOI:** 10.1371/journal.ppat.1011157

**Published:** 2023-03-02

**Authors:** Yannik Voß, Severina Klaus, Julien Guizetti, Markus Ganter

**Affiliations:** Center for Infectious Diseases, Heidelberg University Hospital, Heidelberg, Germany; Joan and Sanford I Weill Medical College of Cornell University, UNITED STATES

## Abstract

Malaria remains a significant threat to global health, and despite concerted efforts to curb the disease, malaria-related morbidity and mortality increased in recent years. Malaria is caused by unicellular eukaryotes of the genus *Plasmodium*, and all clinical manifestations occur during asexual proliferation of the parasite inside host erythrocytes. In the blood stage, *Plasmodium* proliferates through an unusual cell cycle mode called schizogony. Contrary to most studied eukaryotes, which divide by binary fission, the parasite undergoes several rounds of DNA replication and nuclear division that are not directly followed by cytokinesis, resulting in multinucleated cells. Moreover, despite sharing a common cytoplasm, these nuclei multiply asynchronously. Schizogony challenges our current models of cell cycle regulation and, at the same time, offers targets for therapeutic interventions. Over the recent years, the adaptation of advanced molecular and cell biological techniques have given us deeper insight how DNA replication, nuclear division, and cytokinesis are coordinated. Here, we review our current understanding of the chronological events that characterize the unusual cell division cycle of *P*. *falciparum* in the clinically relevant blood stage of infection.

## Introduction

Cell division is a precisely orchestrated sequence of molecular events and has been studied in great detail in many eukaryotic model organisms. Typically, cells proliferate by duplicating their genome and subsequently dividing in two daughter cells, but deviations from this scheme are commonly found on many branches of the eukaryotic tree of life. Unicellular apicomplexan parasites, such as *Toxoplasma* or *Plasmodium*, represent one of those divergent branches, where the cell cycle and its regulation remain enigmatic [1]. Although these parasites use some conserved cell cycle factors [2], the cytoskeletal elements, membrane topologies, and regulatory pathways are organized in a substantially different manner [3,4]. Here, we focus on the malaria-causing parasites of the genus *Plasmodium*, which still cause the death of over 600,000 people per year [5]. Extensive *Plasmodium* proliferation inside erythrocytes causes all malaria-related pathology and generates high numbers of progeny by an unconventional cell cycle mode, termed schizogony.

Among the most important differences to the cell cycles of model eukaryotes are multinucleated cells with asynchronous nuclear divisions, the atypical structure of the centrosome, a specialized daughter cell segmentation mechanism, and the apparent absence of most canonical cell cycle checkpoints. In this review, we lay out our current understanding and knowledge gaps about the chronology of events, starting from the initiation of the first DNA replication during S–phase to the completion of cellularization during cytokinesis.

### Defining entry into the schizont stage

The proliferative cycle begins when a *Plasmodium* merozoite invades a host erythrocyte. Inside, the merozoite transforms into a ring–stage parasite and subsequently into a trophozoite. During this time, the parasite substantially remodels the host cell and consumes its cytoplasm [6–8]. In analogy to the canonical cell cycle (consisting of a gap phase, G1; S–phase, during which DNA is replicated; a second gap, G2; and M–phase, when mitosis occurs), both ring and trophozoite resemble cells in the G1 phase. The trophozoite stage is followed by the schizont stage, during which nuclei multiply. Schizogony is concluded with cellularization and release of the daughter cells. Integrating external stimuli [9], a fraction of asexually proliferating parasites commits to sexual development and form gametocytes, which can continue life cycle progression when taken up by a mosquito. This commitment occurs 34 to 38 hours post invasion (hpi) and is regulated by the transcription factor *Pf*AP2–G [9–11]. Conditional expression of *Pf*AP2–G results in approximately 90% conversion to sexual stages [12], which cease to proliferate.

Classically, the distinction between the trophozoite stage and the schizont stage has been made morphologically, and a schizont is often referred to as a multinucleated cell, i.e., a cell with more than two nuclei. While this classification seems intuitive, it appears artificial from a cell cycle point of view. According to the morphological definition, DNA replication and nuclear division occur in both the trophozoite and the schizont stage. Moreover, intrinsic and extrinsic perturbations appear to demarcate a major cell cycle transition at the beginning of the first S–phase (Box 1) [13–16]. While the classic, morphology-based staging is experimentally easy to accomplish and sufficient for many research questions, it bears limitations as it pools stages that resemble cells in G1 with stages in which S–phase or nuclear division already occurred. Therefore, we encourage, if possible, the use of an alternative, cell cycle–based staging, in particular when investigating the cell cycle of *P*. *falciparum*. Thus, in the context of this review, we consider a schizont as all developmental stages from the onset of the first S–phase to the conclusion of merozoite formation.

**Box 1. Cell cycle checkpoints**

Canonical cell cycle checkpoints are opportunities for the cell to induce a controlled arrest of cell cycle progression until all prerequisites for the next step are met. An example is the spindle attachment checkpoint during M–phase, which ensures that all kinetochores are properly attached to the spindle microtubules, so sister chromatids can be faithfully segregated [109]. Importantly, cell cycle checkpoints share certain characteristics. A checkpoint must be reproducibly induced by a defined error at a defined time but should also be reversible once problems have been resolved. To conclusively demonstrate the existence of a checkpoint, one must further demonstrate that a deletion of a candidate protein prevents checkpoint activation.

Even though recently a first cell cycle checkpoint protein candidate, a potential homologue of Mad1, has been identified in the *P*. *berghei* genome [56], experimental data still argue against the existence of a spindle attachment checkpoint. Indeed, schizonts continue to replicate their DNA in the presence of drugs that interfere with microtubule formation, which causes erroneous nuclear division [110–112]. In contrast, inhibition of DNA replication appears to induce an arrest without readily detectable attempts to divide the nucleus [113].

In line with this, more evidence starts pointing towards a controlled entry into the schizont stage. Deprivation of the essential amino acid isoleucine prior to the onset of S–phase dramatically affects cell cycle progression [13,16]. As the only amino acid absent from human hemoglobin, *P*. *falciparum* largely depends on import of isoleucine from the serum or medium. In the absence of exogenous isoleucine, *P*. *falciparum* development arrests at the trophozoite/schizont transition, which is reminiscent of a controlled entry into S–phase via a G1/S checkpoint. More recent results suggest that the arrest is not complete and that parasites progress through the cell cycle extremely slowly and eventually start DNA replication [16]. Importantly, isoleucine deprivation after S–phase onset does not stall the cell cycle [16], which agrees with a potential checkpoint. Moreover, the phosphorylation status of *Pf*eIF2α depends on isoleucine availability and, although this does not seem to be involved in the developmental delay, it demonstrates the parasite’s responsiveness to amino acid starvation [13]. Similarly, disruption of polyamine synthesis via DL–α–difluoromethylornithine resulted in a reversible arrest at the trophozoite to schizont transition [15], but it is unclear if both responses are rooted in the same regulatory pathway. Together, these findings indicate that a G1/S checkpoint may exist, which controls entry into the schizont stage.

In contrast, several studies have shown that proper nuclear multiplication or orderly segmentation are neither required for organellar development nor for the initiation of egress [14,43,61,90,97]. Thus, there may not be a cytokinesis checkpoint, which would prevent cell cycle progression even if nonviable daughter cells are formed.

Conclusive evidence for cell cycle checkpoints in asexual *Plasmodium* blood stages will require a combination of cell cycle perturbation, time-resolved data acquisition, and genetic deletions. In addition, how the cell cycle and potential checkpoints may be affected by the parasite’s intrinsic circadian clock and the daily host rhythms remains elusive [114].

### The first round of DNA replication

**Fig 1:**
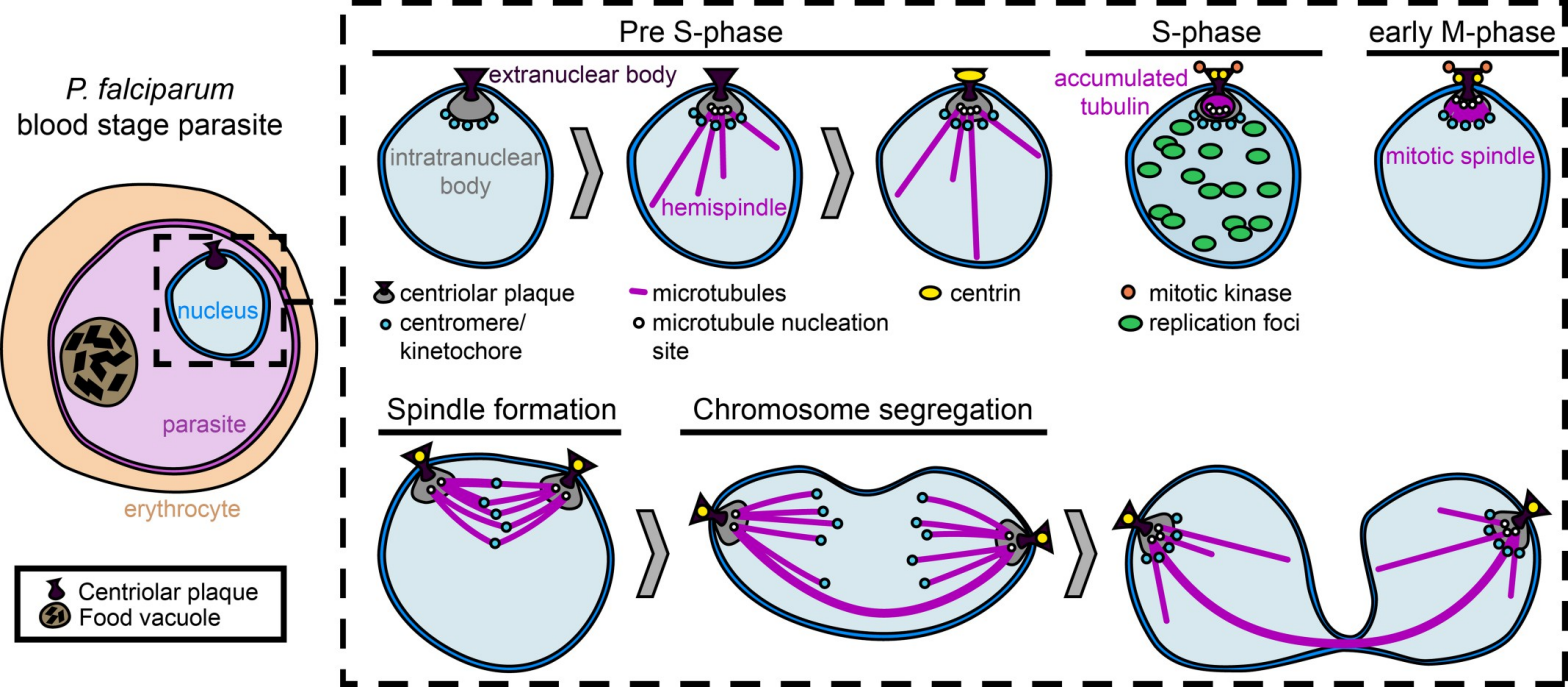
Progression of nuclear cycle stages in mononucleated parasites. Nuclear division is organized around the centriolar plaque, which spans the nuclear envelope and consists of an extranuclear compartment and an intranuclear compartment acting as the microtubule organizing center. Prior to S–phase initiation and the formation of a mitotic spindle, large, dynamic hemispindles can be observed in the nucleus. Centrins are recruited to the extranuclear compartment of the centriolar plaque during the later hemispindle stages before S–phase occurs. Nuclear division is closed, with the duplicated centriolar plaques eventually moving to opposite sides of the dividing nucleus during chromosome segregation, before nuclei are separated.

Currently, the first cellular feature indicating that a parasite with a single nucleus prepares to enter S–phase and nuclear multiplication is the so-called hemispindle, which consists of intranuclear microtubules that radiate from the parasite’s centrosome, called centriolar plaque, into the nucleoplasm (Fig 1) [17,18]. While it is unclear if the hemispindle serves a specific cellular function or whether it forms due to high local concentrations of tubulin [18–20], its presence likely marks the transition from trophozoite to schizont stage and heralds the impending first round of DNA replication [14–16].

In *P*. *falciparum*, various techniques have been used to determine the timing of the first S–phase relative to merozoite invasion. Doubling of fluorescent signals from telomeric repeats stained by in situ hybridization was used as proxy for DNA replication and reported it to start approximately 24 to 26 hpi [21]. However, other studies used flow cytometry with nucleic acid dyes and reported increasing DNA contents approximately 30 to 32 hpi [14,22]. Concordantly, labelling nascent DNA in synchronized parasite cultures reported that the first S–phase commences approximately 31 hpi [23] and incorporation of radioactively labeled nucleotides suggested an onset of DNA replication at approximately 30 hpi [24]. Collectively, this indicates that in *P*. *falciparum* asexual blood stages the first round of DNA replication commences at approximately 30 hpi.

During the preceding G1 phase of canonical cell cycles, the origins of replications are licensed for DNA replication. This licensing occurs by the sequential binding of the origin of replication complex (ORC) and two minichromosome maintenance (MCM) complexes along with other factors at the DNA. At the transition from G1 to S–phase, cyclin-dependent kinase (CDK) phosphorylates components of the ORC and MCM complexes, more proteins assemble (including the DNA polymerases), and DNA replication commences. At the same time, CDK activity inhibits the licensing of origins, as, e.g., phosphorylated ORC does not bind to DNA. This temporal separation of licensing of origins and origin firing during DNA replication ensures that the DNA is replicated once and only once per cycle [25,26].

Only some of the components needed to reconstitute origin licensing and firing in vitro were identified in the *Plasmodium* genome by sequence homology, but key components like ORC and MCM are conserved [14,27–29]. Kinases related to canonical CDK have been shown to play a pivotal role for DNA replication in the blood stage and in activated male gametocytes [14,30,31]. Depletion of the essential cdc2-related kinase (*Pf*CRK) 4 led to a reversible arrest just prior to the onset of DNA replication [14]. In absence of *Pf*CRK4, several components of the DNA replication machinery are less phosphorylated, including components of ORC and MCM complex, DNA polymerase alpha and epsilon, as well as topoisomerase 2. *Pf*CRK4 activity is required for the initial and subsequent rounds of DNA replication. The serine/arginine–rich protein kinase (*Pf*SRPK) 1 likely acts downstream of *Pf*CRK4, and deletion of *Pf*SRPK1 led to a reduced number of daughter cells and defective male gamete development in the mosquito vector [14,32]. A potential role for the related kinase *Pf*CRK5 for DNA replication in schizonts was recently questioned [31,33], but CRK5 appears to fill the role of *Pf*CRK4 during DNA replication in activated male gametocytes, in both *P*. *falciparum* and *P*. *berghei* [30,31]. These regulators offer a starting point to identify other molecular determinants and characterize the underlying regulatory networks that govern the entry into the schizont stage.

The dynamics of the active DNA replication fork were recently determined by pulse-labeling of nascent DNA. This was made possible by ectopic expression of a viral thymidine kinase that phosphorylates nucleoside analogues into nucleotides, which can be incorporated into nascent DNA and later be detected by immunofluorescence or click chemistry [34]. In early schizonts of both *P*. *falciparum* and *P*. *knowlesi*, DNA is replicated at approximately 1.5 kb per minute and this rate decreased over time to approximately 1 kb per minute in late schizonts. At the same time, the spacing of two origins of replication also decreases. The reasons for this slowdown are not known, but nucleotide availability or a changing chromatin state may play a role [23,35].

### Coordination of DNA replication and nuclear division

**Fig 2:**
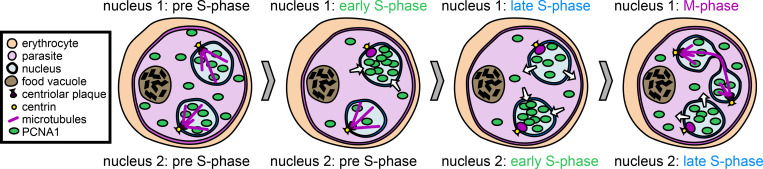
Nuclei cycle asynchronously in multinucleated parasites. After nuclear division, hemispindle structures remain briefly within the separated nuclei. In parasites with two nuclei, one may enter S–phase earlier than the other; S–phase entry is marked by the rapid accumulation of the replication marker PCNA1 within a nucleus. Asynchrony is visible in both S–phase and nuclear division, as nuclei may show nuclear accumulation of PCNA1 as well as different microtubule structures at different times.

Once DNA replication is initiated, it takes approximately 15 hours in *P*. *falciparum* until the next generation of parasites are produced by repeated rounds of DNA replication and nuclear division [23,36]. Two different models for the coordination of DNA replication and nuclear division in a *Plasmodium* schizont have been proposed. One model assumed that several rounds of DNA replication occur prior to nuclear divisions, thus forming a polyploid nucleus [27,37]. This proposed mode is similar to what has been described for activated male gametocyte, which undergoes three rounds of DNA replication. Thereby, the DNA content of the single nucleus increases from 1 to 8 *C*, and, subsequently, this nucleus is divided and male gametes emerge [38–40]. The other model proposed consecutive and alternating rounds of DNA replication and nuclear division [21,41,42]. This model assumes that the DNA content of a given nucleus does not exceed the diploid level and that the DNA content and the number of nuclei in a given parasite increase gradually. Experimental data strongly support the latter mode, i.e., consecutive rounds of DNA replication and nuclear division (Fig 2) [23,36], and, to our knowledge, polyploid nuclei have only been described in mutant parasites, such as the *Pf*MCMPB knockdown [43].

### The first nuclear division

A central regulatory hub for nuclear division is the centrosome as it organizes microtubules into a mitotic spindle that drives segregation of the replicated chromosomes. Unlike in mammalian cells or in the related apicomplexan parasite *Toxoplasma*, the centriolar plaque of asexual *Plasmodium* spp. blood stages does not contain centrioles [44]. It was previously observed merely as an amorphous electron dense mass embedded in the nuclear envelope [45]. Recent work showed that the centriolar plaque of asexual blood stages is a protein-dense structure consisting of an extra- and an intranuclear compartment. The latter is devoid of chromatin, but microtubule minus ends are nucleated here [18]. Both compartments of the centriolar plaque are connected via an opening in the nuclear envelope, which is densely filled with protein and may be a derivative of a nuclear pore (Fig 1) [18,46]. This overall organization of the centriolar plaque is also found in sexual stages, although they contain an extranuclear centriole-like structure [47,48].

Four centrins are among the few canonical centrosomal proteins that are conserved in *Plasmodium* spp. [18,48–50]. Centrins accumulate at the centriolar plaque when the hemispindle is already present, and although being essential for blood stage parasites, their precise function remains unknown (Fig 1, Table 1) [50,51]. Another protein of the centriolar plaque is the essential anaphase promoting complex subunit 3 (APC3) of the related parasite *P*. *berghei* [52]. The canonical anaphase promoting complex/cyclosome is an E3 ubiquitin ligase that promotes cell cycle progression [53]. In activated male gametocytes, *Pb*APC3 is involved in chromosome condensation and cytokinesis, but its function for *P*. *falciparum* schizogony remains unknown [52].

**Table 1:** Selected marker for live–cell imaging of *P*. *falciparum* schizogony in blood stages.

Structure	Target	Fluorophore/Dye	Source
Centriolar plaque (extranuclear compartment)	Cen3, Cen4, Cen1	GFP	[18,48,50,93]
Centriolar plaque (intranuclear compartment)	γ–Tubulin	GFP	[52]
Centromere	CenH3	GFP	[54]
Kinetochore	NDC80, NUF2, SKA2	mCherry, GFP	[48,55,56]
DNA	5–SiR–Hoechst dye	SiR–Hoechst dye	[18]
Nucleoplasm	NLS–mCherry	mCherry	[36,140]
Heterochromatin/Telomeres	HP1	GFP	[141]
Active DNA replication	PCNA1	GFP	[36]
Nucleopores	Nup313/138/221	GFP	[18,142]
Microtubules	SPY555–Tubulin dye,	SPY555 dye	[18]
Microtubule	Kinesin8X, Kinesin–5, EB1	GFP	[48,117,143]
Membrane (e.g., nuclear envelope)	Lipids	BODIPY TR Ceramide	[46,116]
IMC	GAP45, MAL13P1.130, PFE1285w, GAP50, ALV5	GFP, mCherry	[92,93,144]
Basal complex	BTP1, MORN1, HAD2,	GFP	[94,145]
(Merozoite) plasma membrane	SMS1	mCherry	[94]
Rhoptry	ARO, RON12, AIP	mCherry	[144]

The centromeres, as marked by histone *Pf*CenH3, cluster at the nuclear periphery and are positioned at the intersection between the centriolar plaque and chromatin (Fig 1) [18,54]. In *P*. *falciparum* and *P*. *berghei*, the kinetochore marker NDC80 is recruited to the centromeres during the trophozoite stage, potentially reflecting the assembly of the kinetochore [48,55]. Our understanding of the *Plasmodium* kinetochore composition was recently expanded through the discovery of previously unknown kinetochore proteins and their organization in the multilayered kinetochore, with four individual compartments [56]. In addition, the phosphatase PP1 of *P*. *berghei* partially colocalizes with kinetochores from the trophozoite stage until egress. Inducible depletion of *Pf*PP1 at the ring stage led to decreased DNA replication dynamics, which most likely translate into fewer daughter cells. Later depletion did not affect DNA replication dynamics, but when the phosphatase was absent from the trophozoite stage onward, schizonts had a lower number of nuclei. In addition, depletion of *Pf*PP1 at any stage of the intraerythrocytic development revealed a critical role for this phosphatase during egress. The molecular mechanism through which *Pf*PP1 affects cell cycle progression remains, however, elusive [57,58].

Likely at the onset of S–phase, the hemispindle rearranges into an immature mitotic spindle (Fig 1) [18,36]. At the same time, the centrin signal duplicates [18], which coincides with the accumulation of the essential mitotic aurora kinase *Pf*Ark1 at the centriolar plaque (Fig 1) [59]. Treatment with the aurora kinase inhibitor hesperadin suggested a role for *Pf*Ark1 in spindle formation and nuclear division, potentially through impaired centriolar plaque function [60]. After S–phase has concluded, there is a lag period before the nucleus divides [36]. Likely during this time, the mitotic spindle completes its bipolar arrangement and kinetochore microtubules attach to the duplicated chromosomes (Fig 1). The *Plasmodium* spindle is initially highly compact with an average length of approximately 550 nm until it rapidly extends to segregate both chromosome sets (Fig 1) [18,46]. How the sister chromatids are released, what generates the necessary forces to drive them apart, and how kinetochore and interpolar microtubules are reorganized are only some of the open questions around anaphase in *P*. *falciparum*. Ultimately, nuclear division is completed by karyofission [18,46].

Few factors have been functionally associated with nuclear division, but depletion of *Pf*MCMBP led to uneven or incomplete karyofission, potentially through defective chromosome segregation [43,46]. Similarly, deletion of schizont egress antigen–1 (*Pf*SEA–1), which localizes close to the centromeres, led to severe defects in DNA segregation and nuclear division [61].

The intermediate steps of mitotic spindle assembly and nuclear division and the regulatory proteins, such as the spindle microtubule associated *Pf*EB1 [48], remain to be characterized in more detail. Recent advances in imaging techniques now permit to resolve nuclear division with sufficient spatial and temporal resolution (Box 2).

**Box 2. Advances in microscopy promoting the study of *Plasmodium* proliferation**

*Time lapse microscopy*. The difficulty to tightly synchronize blood stage malaria parasites, partly due to missing checkpoints, is significantly limiting the study of schizogony using bulk population analysis. Understanding the dynamic progression through schizogony and following the fate of individual nuclei requires temporally resolved single cell data. Time-lapse fluorescence microscopy has been pioneered in the liver stage development [115] and was used to follow protein transport in asexual blood stages [116]. 4D live–cell imaging has only been more recently applied to study nuclear multiplication [36] and to follow the dynamics of the microtubule associated protein *Pb*kinesin–5 [117]. Time-lapse imaging of microtubules has been achieved through live–cell compatible fluorogenic microtubule dyes and image deconvolution [18]. The list of live–cell markers for schizogony is, however, steadily growing (Table 1). As the generation of transgenic parasite lines is still a rate-limiting step, the use of state-of-the-art live–cell dyes is an important tool [118]. Due to their excellent background-to-noise ratio, fluorogenic dyes have significantly contributed to this progress [119,120], while classical dyes like the membrane label BODIPY still have their utility [46,116]. The observation that, e.g., Hoechst-based dyes can impact mitotic progression, however, calls for careful monitoring of their effect on cell physiology [18]. As recently demonstrated, 4D multicolor live–cell microscopy generates large quantitative data sets that can inform mathematical modelling to generate insightful predictions and hypotheses about schizogony [36]. The key challenge of time lapse imaging remains to balance spatial and temporal resolution with phototoxic effects. In this respect, the development of lattice light–sheet microscopy [121] has been a milestone that has showcased its exceptional potential in the analysis of merozoite invasion [122], and we await its application in the study of schizogony.

*Super-resolution microscopy*. The second important challenge in studying schizogony is the small size of the nucleus and associated mitotic structures. Structure–illumination microscopy (SIM) was the first super-resolution microscopy technique adapted to parasites to investigate merozoite invasion [123,124]. It offers a 2-fold improved resolution in 3D and can, despite stronger illumination, be used on live parasites [125]. A significantly higher resolution can be achieved by Stimulated Emission Depletion (STED) nanoscopy [126], which has been used in liver stage parasites [127]. Two approaches, namely RescueSTED and Guided STED, have made STED available for blood stages by avoiding the destructive hemozoin illumination by the STED laser [128,129] and thereby contributed to building the current working model of the centriolar plaque (Fig 1) [18].

Although STochastic Optical Reconstruction Microscopy (STORM) has been successfully used to characterize the subdiffraction organization of the adhesion structures that parasites assemble on the surface of red blood cells [130,131], its application remains technically challenging and has limitations for live, 3D, and multicolor applications.

Lately, a new type of super-resolution microscopy based on the isotropic expansion of labelled cells in a gel, called expansion microscopy (ExM), has gained more attention [132]. The cellular expansion by a factor of 4 to 5 allows multicolor imaging of full 3D–stacks of fixed cells while reaching a resolution similar to STED. A later optimization, named ultrastructure expansion microscopy (U–ExM) [133], has been applied in multiple parasite life cycle stages to reveal organization of cytoskeletal as well as intranuclear microtubules with stunning detail [18,100]. Combination of U–ExM with NHS–Ester staining (pan–ExM) reveals a protein density map of the entire cell [46,100].

Nevertheless, maximal spatial resolution is still achieved by electron microscopy (EM), which has contributed the key initial insight into schizogony in early transmission EM studies [45,134–136]. In later years, 3D electron EM technologies have become more important, such as the highly resolving tomography [18,137] and focus ion beam scanning electron microscopy (FIB–SEM), which can produce 3D reconstruction of entire cells, albeit with lower resolution [95,97,138].

The benefits of fluorescence and electron microscopy have now been combined in *Plasmodium* using correlative light and electron microscopy (CLEM) approaches that can put specific protein labelling in the ultrastructural context acquired in EM [18,36,139]. CLEM approaches are, however, still in their infancy in the *Plasmodium* field, likely related to their challenging implementation. Ultimately, all aforementioned techniques rely on consistent cellular markers, antibodies, and sample preparation [128]. While for most relevant division structures reliable markers are known (Table 1), a genetically encoded nuclear envelope marker is still lacking [46].

### Asynchronous nuclear cycles in multinucleated schizonts

In contrast to many other multinucleated cells, such as the early *Drosophila* embryo [62] or the unicellular marine eukaryote *Sphaeroforma arctica* [63], the successive rounds of DNA replication and nuclear division in *Plasmodium* schizonts occur asynchronously (Fig 2) [18,23,36,64].

Asynchronous nuclear divisions can also be observed in the hyphae of the multinucleated fungi *Ashbya gossypii* [65]. Here, asynchrony is dependent on cytoplasmic territories that are established by the nuclei and which limit the diffusion of cytoplasmic factors that regulate nuclear cycle progression. In addition, nucleus-intrinsic factors, such as different transcriptional activity, contribute to the asynchronous nuclear division [66–69]. Compared to *Ashbya*, where nuclei are on average approximately 5 μm apart, *P*. *falciparum* nuclei are typically in much closer proximity, sometimes less than 100 nm apart [36,69]. This suggest that nucleus intrinsic mechanisms probably contribute more to asynchrony than cytoplasmic ones. However, the underlying molecular mechanisms that establish asynchronous nuclear cycles in *Plasmodium* spp. remain elusive.

To obtain a molecular understanding of the underlying mechanisms, two recent studies investigated the dynamics of nuclear multiplication in the schizont stage. One employed pulse labelling of nascent DNA in thymidine kinase–expressing *P*. *falciparum* and *P*. *knowlesi* parasites [23]. The other study used live–cell imaging of a *P*. *falciparum* nuclear cycle sensor line, which expresses the red fluorescent protein mCherry in all nuclei and a component of the DNA replication fork, the proliferating cell nuclear antigen (*Pf*PCNA) 1 fused to GFP [36]. Since *Pf*PCNA1 accumulates only in S–phase nuclei that replicate their DNA, this dual-color line allows tracking of nuclear cycles in analogy to the fluorescence ubiquitination cell cycle indicator (FUCCI) for mammalian cells (Fig 2, Table 1) [70].

Both studies report a high cell-to-cell variability and directly showed that not only nuclear division occurs asynchronously, but also DNA replication. Live–cell imaging also showed that the asynchrony between sister nuclei is predominantly introduced during the time from nuclear division to the ensuing S–phase, which may be similar to the G1 phase of the canonical cell cycle (Fig 2) [36].

While both studies report similar dynamics in early schizonts (e.g., approximately 50 minutes for the initial round of DNA replication), the datasets are less coherent for late schizonts. From different regimens of pulse labelling, it was concluded that the time between consecutive DNA replications increases as nuclear multiplication progresses, while the time needed for DNA replication decreases. In contrast, live–cell imaging suggested that S–phases in late schizonts, where more nuclei are in S–phase at the same time, become longer due to competition for shared limited resources.

Mathematical modelling showed that schizonts need to slow down their nuclear cycle dynamics over time to produce the experimentally determined final number of nuclei [36]. This overall slowing can be achieved in different ways. While live–cell imaging suggests slower DNA replication as more nuclei replicate simultaneously [36], nucleotide analogue labelling indicated an extended (≥5 hours) replicative arrest of one to two nuclei per cell [23]. Another open question concerns the end of nuclear multiplication and whether or not nuclei undergo a final synchronous round of DNA replication. While pulse labelling supports a final synchronous round [23], live–cell imaging and mathematical modelling suggest that nuclei cease to multiply without a final synchronous round of DNA replication [36].

When comparing *P*. *falciparum* and *P*. *knowlesi*, the overall pattern of nuclear multiplication and replication fork velocity was highly similar. But although both species spent approximately 30% of their blood stage cycle multiplying their nuclei and have a comparable genome size, the dynamics of nuclear multiplication differed and the gap phases between successive S–phases were shorter in *P*. *knowlesi* than in *P*. *falciparum* [23].

Tracking nuclei until the end of schizogony to see if the final round of DNA replication is distinct from previous rounds and to see if some nuclei undergo replicative arrest will help resolve some of these conflicting data and further inform on how nuclear cycles are organized during nuclear multiplication. In any case, asynchronous nuclear cycles appear to support rapid parasite proliferation in an environment with limited resources [36]. An open question is how transcriptional homeostasis is maintained as nuclei multiply and the cellular DNA content increases [71].

### Concluding nuclear multiplication

An essential decision for the parasite is when to stop nuclear multiplication and transition to cell division—in other words, how does the parasite know when the desired number of daughter nuclei is reached? In general terms, this decision may be accomplished by two different mechanisms [72,73]: A timer mechanism would stop further nuclear multiplication after a predefined time period elapsed. Conversely, a counter mechanism would stop further multiplication once a predefined number of nuclei is reached. Timer and counter mechanisms can be experimentally distinguished by correlating parameters such as the length of the initial cycles, the length of the overall nuclear multiplication time, and the final number of nuclei. Correlating the duration of the initial nuclear cycle with the overall time spend for nuclear multiplication strongly supports a counter mechanism [36], but how this counter could be set remains elusive.

Parasites cultured in the same conditions display a remarkable variability in progeny numbers, ranging from less than 10 to more than 30 [73–77]. This suggests that factors intrinsic to the host–parasite unit may play an important role. Indeed, when comparing normal to hemoglobinopathic host erythrocytes, the number of daughter cells correlated positively with the mean erythrocyte volume and hemoglobin content [77]. However, whether this correlation is entirely due to cell size or is also influenced by the molecular characteristics of each specific type of haemoglobinopathy remains to be seen.

To better understand the counter mechanism, it will also be critical to investigate how environmental factors that act on the entire parasite population affect the number of daughter cells, such as nutrient levels in cell culture media or patient blood plasma [78–81].

### Organellar genome replication

Aside from the chromosomal DNA contained within the nucleus, *Plasmodium* harbors two additional organelles, which carry their own genome: the apicoplast, which is specific for Apicomplexa, and the mitochondrion. Both must be distributed to the daughter cells during schizogony, which requires replication of their genomes. The apicoplast originated from a secondary endosymbiosis event and is involved in several essential metabolic processes, such as isoprenoid and fatty acid synthesis [82]. The AT-rich 35-kB circular apicoplast genome is present in no more than three copies per parasite in early stages, and its replication was estimated to start shortly before the onset of chromosomal replication and then continues in parallel to DNA replication in the nucleus [83]. Replication is dependent on the essential multifunctional plastidic DNA replication/repair enzyme complex (PREX). This complex is encoded in the nuclear genome and carries primase, helicase, and polymerase domains [84]. Other replication factors such as gyrases and single-strand-binding proteins (SSB) are likewise encoded in the nuclear genome and are imported into the apicoplast for replication and repair [84].

In contrast, the highly conserved 6 kB mitochondrial genome is present in approximately 20 copies per mitochondrion in *P*. *falciparum* [85]. In the mitochondrion, the genomes are arranged into highly complex linear concatemer structures, which are replicated at approximately the same time as the chromosomal DNA [85]. Like for the apicoplast genome, replication of the mitochondrial genome depends on nuclear-encoded proteins, such as the *P*. *falciparum* topoisomerase III [86].

A *P*. *falciparum* mutant, in which nuclear DNA replication is profoundly suppressed, shows seemingly well-developed apicoplasts and mitochondria [14]. Thus, organelle development and potentially organellar DNA replication may be independent of chromosomal replication. Quantification of the organellar DNA content under these circumstances will inform on if and how apicoplast and mitochondrial DNA replication are coupled to cell cycle regulation and chromosomal replication.

### Preparing for daughter cell formation

**Fig 3:**
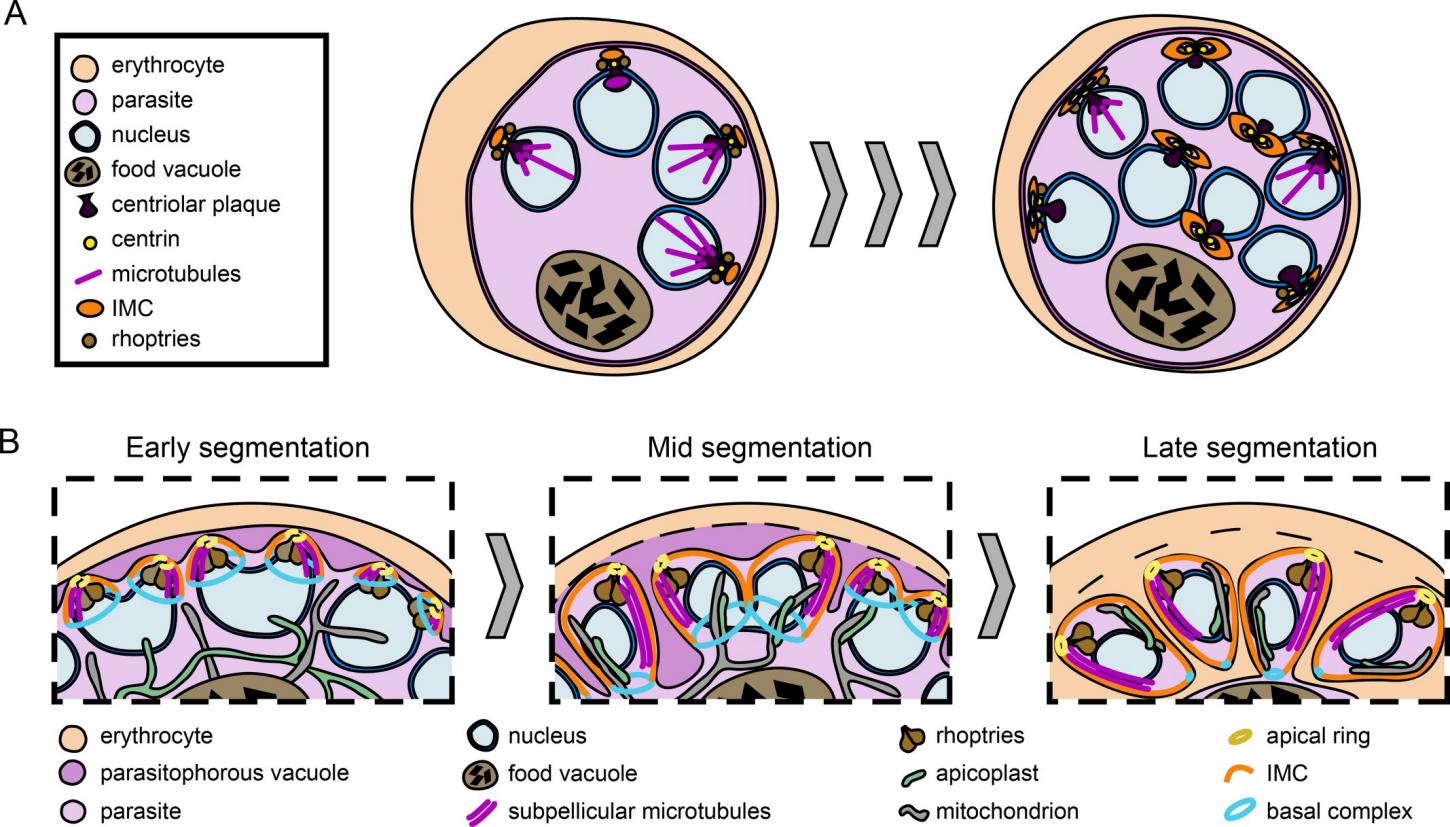
The end of nuclear multiplication and formation of daughter cells. **(A)** Structures and organelles involved in cytokinesis start forming during early nuclear cycles. Proteins of the IMC and the rhoptries start organizing around the centriolar plaque in cells with nuclei numbers as low as four and develop into larger structures as nuclear multiplication progresses. **(B)** Daughter cells are formed during segmentation. Concomitant to daughter cell formation, the nuclei are divided in a final, semisynchronous round of nuclear division. One pair of rhoptries associates with the apical end of the developing daughter cells. Segmentation commences when the plasma membrane (PM) starts invaginating. During segmentation, the IMC, which sits below the PM and originates from the apical end, starts enveloping the emerging daughter cell alongside the PM, with the basal complex localizing to the leading edge of the IMC. Concurrently to the expansion of the IMC, subpellicular microtubules start developing from the apical end of the emerging daughter cells. At the end of segmentation, the daughter cells now containing all necessary organelles are pinched off a remnant body containing the food vacuole.

While the nuclei multiply, many organelles are already being primed for daughter cell formation [87,88]. The apicoplast and mitochondrion grow into large, branched structures as their genomes are replicated, and the ER becomes a complex structure that extends throughout the cell [89]. Already in schizonts with as little as three nuclei, first components of the inner membrane complex (IMC) can be detected [90]. The mature IMC is a system of large, flattened vesicles that reside directly underneath the plasma membrane of merozoites and is a crucial part of the parasites’ pellicle [91]. In schizonts, the developing IMC initially has a cramp-like shape, and in later schizonts, the IMC is an ellipsoid disk with two small holes developing adjacent to the centriolar plaque. Subsequently, the IMC is pulled around the nascent daughter cell by the basal complex, which acts as a contractile ring [92–95]. Similar to the IMC, rhoptries also start developing adjacent to the centriolar plaque in schizonts with as few as four nuclei [49]. Thus, the centriolar plaque may direct the positioning of the apical complex and structures that are critical for cytokinesis and invasion, which begin their development early during nuclear multiplication. Being important for both processes, the centriolar plaque may connect nuclear multiplication and organization of cytokinesis (Fig 3A).

### Initiation and progression of segmentation

Daughter cell formation, cellularization, or segmentation are terms that describe the same overall process, i.e., the transition from a single multinucleated cell into multiple daughter cells with a single nucleus each (Fig 3). After the final DNA replication, it is assumed that nuclei arrest in the diploid stage until the final nuclear division occurs concomitantly to segmentation. Early in this process, the parasite plasma membrane (PM) begins to invaginate [95], and the basal complex localizes to the leading edge of the PM, pulling the IMC along. PM and IMC encapsulate the emerging daughter cells starting from the apical end until the basal complex comes to rest at the posterior end. Then, the daughter cells are pinched off the residual body, which contains the food vacuole of the mother cell (Fig 3B) [94–97]. With the expansion of the IMC, subpellicular microtubules assemble from the apical ring, supporting the IMC [98–100]. Why *P*. *falciparum* merozoites contain only one or two subpellicular microtubules while *P*. *berghei* merozoites contain approximately nine microtubules is unclear [100].

Contrary to previous hypotheses [1], nuclear division during segmentation does not seem to occur in perfect synchrony (Fig 3B) [95]. At early stages, most nuclei associate with two sets of rhoptries instead of one. Simultaneously, a small number of nuclei may associate with four sets of rhoptries, indicating a certain degree of asynchrony or imprecision. In each set of rhoptries, one rhoptry appears more mature than the other. All rhoptry necks reach into the apical ring, which is not yet well defined during early stages of cellularization. At this point, the apicoplast and the mitochondrion are still large, branched structures that wind independently around the nuclei (Fig 3) [95]. During mid-segmentation, the parasites’ PM is strongly invaginated at some places, and about half of the nuclei have one set of rhoptries, while the other half is still associated with two sets, again indicating a certain degree of asynchrony. At mid-segmentation, the apicoplast is already divided, while the single mitochondrion has still a branched structure. At this stage, both organelles are now closely associated with each other [89,95]. Only during the final steps of segmentation, the mitochondrion is also divided and, along with the nucleus and an apicoplast, packaged into the emerging daughter merozoite [89,95]. At some point before daughter cells are completely separated, the ER fragments and forms crescent organelles that associate with each daughter cell [89].

The distribution of the apicoplast to the daughter cells depends on *Pf*actin 1, which is also involved in apicoplast branching [101,102], but the molecular basis for mitochondrial fission and how it is coordinated with cytokinesis are not well understood [87]. The only investigated mitochondrial fission protein *Pf*Fis1 is not essential, although the *P*. *falciparum* protein kinase 7 may be involved in regulation of mitochondrial genes involved in fission [103,104].

*Pf*actin 1 and the actin nucleator *Pf*formin–2 are also required for proper and efficient segmentation [101,102] as is the cyclin H homolog *Pf*Cyc1, which potentially acts in concert with the CDK-activating kinase assembly factor *Pf*MAT1 and the Cdk7 homologue *Pf*MRK [105]. Cells lacking *Pf*Cyc1 fail to form merozoites, although nuclei and apical organelles appear normal [105]. Additionally, parasites with a defective IMC, e.g., in absence of the apical protein *Pf*MOP, fail to form daughter cells properly [90]. Similarly, depletion of *Pf*MCMBP or deletion of *Pf*SEA–1 led to defective nuclear division, which translated into abnormal merozoite formation. Still, segmentation and egress were induced in absence of *Pf*MCMBP or *Pf*SEA–1, and a fraction of the respective mutant merozoites lacked the nucleus entirely [43,61].

The basal complex and, specifically, the protein *Pf*CINCH have been shown to facilitate separation of merozoites from the residual body [97]. Another basal complex protein, *Pf*MORN, is dispensable for blood stage development, including cytokinesis [106]. At the time of their release from the residual body, merozoites contain a single pair of rhoptries, and egress from the host erythrocyte is already initiated, as the parasitophorous vacuolar membrane is being disintegrated (Fig 3) [95]. Egress concludes by rupture of the host erythrocyte plasma membrane, and mature merozoites are released into the blood stream to start a proliferative cycle anew [107,108].

Daughter cell segmentation is a highly complex process that should be precisely controlled to allow for the efficient formation of viable daughter cells. Further improvements in live–cell imaging will help elucidate the regulatory cascades that initiate apical complex formation and organelle distribution, and how they relate to nuclear multiplication.

## Conclusions

Although studying the cell cycle of *Plasmodium* (re)gained momentum in the last years, it remains enigmatic. Resolving conflicting data, employing modern biology approaches, and asking fundamental questions will help us to gain more insight into the molecular and cellular mechanisms that orchestrate *Plasmodium* proliferation. As we are just beginning to understand proliferation of *Plasmodium* in the blood stage, more challenges and discoveries await when proliferation in the oocyst and the liver stage will be in focus. These insights are a prerequisite to unlock the therapeutic potential of the parasite’s unusual cell cycle. In addition, understanding *Plasmodium* proliferation will also inform on the diversity of cell cycle modes and on the fundamental biology of an early branching eukaryote.
